# Trends and disparities in thyroid cancer related mortality from 1999 to 2023: a CDC WONDER database analysis

**DOI:** 10.1186/s12902-026-02402-y

**Published:** 2026-07-07

**Authors:** Reesha Yadav, Christopher Bine, Jessica Ung, Olivia Foley, Abubakar Tauseef

**Affiliations:** 1 Creighton University School of Medicine, 7500 Mercy Rd, Omaha, NE 68124 USA; 2 Department of Internal Medicine, Creighton University School of Medicine, 7500 Mercy Rd, Omaha, NE 68124 USA

**Keywords:** Thyroid cancer, Mortality trends, Epidemiology, United States, Public health, Disparities, Endocrine neoplasms, Population-based analysis

## Abstract

**Background:**

Thyroid cancer mortality has varied across demographic groups in the US, but comprehensive recent analyses are limited. This population-based retrospective cohort study examines trends in thyroid cancer-related mortality from 1999 to 2023 using data from the Centers for Disease Control and Prevention Wide-ranging Online Data for Epidemiologic Research (CDC WONDER).

**Methods:**

Mortality due to malignant thyroid neoplasms (ICD-10 C73) was included. Eligibility included US resident deaths recorded between 1999 and 2023 with thyroid cancer as the underlying cause. Trends in age-adjusted mortality rates (AAMR, per 100,000) across sex, age, race/ethnicity, urbanization, and geographic regions were analyzed using Joinpoint regression with Monte Carlo permutation to estimate annual percent change (APC) and average annual percentage changes (AAPC).

**Results:**

During the study period, 53,145 deaths were attributed to malignant neoplasms of the thyroid. The overall AAMR declined from 0.86 to 0.77 per 100,000. Both sexes showed increased mortality until about 2019 and 2020, following which there were significant declines in mortality. Male patients experienced a higher mortality increase pre-2020 and steeper decline post-2020. Rural populations observed greater mortality increases compared to urban populations. Black or African American patients and White patients had consistently lower thyroid cancer-related mortality than Asian or Pacific Islander and Hispanic patients. Asian or Pacific Islander patients demonstrated relatively stable AAMRs over the study period without a statistically significant change. Older age groups, especially ≥ 85 years, had the highest crude mortality and significant increase in mortality post-2017. The South, Midwest, and West regions had significant mortality increases with a sharp rise in the South post-2019.

**Conclusions:**

Despite an overall decline in malignant thyroid neoplasm related mortality since 1999, there are persistent disparities due to sex, age, urbanization, race and region. Future research should investigate the drivers of the disparities closely, and healthcare policy should also aim to improve availability and accessibility to thyroid cancer related education, screening, detection, and treatment especially to those populations with disproportionately high risk.

**Clinical trial number:**

Not applicable.

**Supplementary Information:**

The online version contains supplementary material available at https://doi.org/10.1186/s12902-026-02402-y.

## Introduction

Thyroid cancer is a significant global health concern. According to GLOBOCAN 2022 estimates, there were over 821,000 new cases of thyroid cancer worldwide in 2022, making it the seventh most common cancer overall and the fifth most common among women. The mortality remains relatively low at an estimated 44,000 deaths globally [1].

According to the National Cancer Institute, thyroid neoplasms currently stand as the 12th most common cancer within the United States (US) [2]. Histologically, thyroid cancer includes several subtypes: papillary (the most common), follicular, medullary, and anaplastic carcinomas, with differing incidence patterns across sex, age, and race [3–5]. Established risk factors include exposure to ionizing radiation as a child [6–10], obesity [11–12], estrogen exposure [13–14], and preexisting thyroid conditions, such as Hashimoto’s thyroiditis [15].

In the US, the incidence and mortality of thyroid cancer have increased. It has been observed that the incidence rate of thyroid neoplasms has increased from 7.32 per 100,000 people in 1999 to a high of 14.92 per 100,000 people in 2014, and a similar incidence rate has since been maintained in the following years [2]. Importantly, mortality trends have demonstrated a similar increase. Kitahara et al. noted that from 1992 to 2012 there was a significant increase in thyroid cancer related mortality with an APC of 0.8% per year [5]. Megwalu and Moon describe that between 2000 and 2018 incidence-based mortality rates rose with an APC of 1.12 [16]. Although improvements in imaging and diagnostic scrutiny may partially explain rising incidence, disparities across demographic groups suggest additional biological, environmental, and healthcare-related contributors [17–18].

In the US, incidence trends differ across racial and ethnic groups. Papillary thyroid cancer increased most rapidly among non-Hispanic whites (APC 5.6% per year) and least among Asians/Pacific Islanders (APC 2.3%), with intermediate increases among Blacks (APC 4.8%) and Hispanics (APC 3.3%) [17]. Prior studies have demonstrated differences in incidence trends and survival outcomes among racial and ethnic populations, potentially reflecting differences in healthcare access, environmental exposures, and other risk factors [17–19]. Because age is an established prognostic factor in thyroid cancer and is incorporated into AJCC staging for differentiated thyroid cancer, this study focused on age-adjusted mortality rates to better account for differences in population age structure over time [20].

Currently, there are few comprehensive studies examining trends in thyroid cancer-related mortality across diverse demographic subgroups. While prior studies have used large databases such as SEER, the CDC WONDER database provides a complementary national mortality dataset with broad demographic coverage [21]. Unlike prior SEER- or incidence-based analyses, this study evaluates age-adjusted mortality trends using CDC WONDER data through 2023 with subgroup analyses across urbanization, race/ethnicity, sex, age, and geographic region. Therefore, the aim of this population-based study was to characterize thyroid cancer-related mortality trends in the United States from 1999 to 2023, with emphasis on temporal patterns across demographic subgroups.

## Methods

### Data collection

This population-based retrospective cohort study examined trends in deaths due to malignant neoplasms of the thyroid gland in the United States from 1999 to 2023. Data for this study were obtained from the Centers for Disease Control and Prevention Wide-ranging Online Data for Epidemiologic Research (CDC WONDER) Database [21]. CDC WONDER database compiles national death certificate information based on International Classification of Diseases (ICD) coding. Institutional Review Board (IRB) approval and informed consent were not required, in accordance with the Common Rule because the data contains no personally identifiable information. This study includes the deaths attributed to malignant neoplasms of the thyroid gland defined by the 10th edition of the International Classification of Diseases (ICD-10) code C73 from the Current Final Multiple Cause of Death Data between the years 1999 and 2023. Pertinent demographic information such as sex (female, male), urbanization (rural, urban), race, ten-year age groups, and geographic location of deaths due to malignant thyroid neoplasms were extracted for analysis. The race category was stratified to Asian or Pacific Islander, Black, Hispanic, and White patients.

While the CDC WONDER database provided mortality rates for most of the subgroups, this study excluded certain subgroups marked to have “unreliable rates” due to the database’s death count for that subgroup being less than 20 [21]. Data for American Indian/Alaska Native patients was excluded as it was reported as unreliable by the CDC WONDER database. The age stratification included the 10-year data from age groups of 35 years old to 85 + years old and excluded the 25–35 year old cohort because the data was determined unreliable. Urbanization data was limited between the years 1999 and 2020 because the database did not include that classification after the year 2020. Demographic characteristics of descendants were summarized using frequencies and percentages. The state distribution was also excluded because the data was unreliable and uneven. While the CDC WONDER database offers comprehensive national coverage, it may be limited by potential misclassification of cause of death and coding changes over time [21].

### Data analysis

The age-adjusted mortality rates (AAMRs) per 100,000 persons were calculated utilizing the year “2000 U.S. standard” via information gathered from the CDC WONDER database. AAMRs represent mortality rates that have been standardized to a reference population to account for differences in age distribution, allowing for fair comparisons across groups and over time. The trends in AAMRs were examined utilizing the Joinpoint Regression Program [22]. Joinpoint regression identifies statistically significant changes in data trend over time by fitting a series of joined log-linear segments. Parameters were automatically set by the software, as all data was analyzed utilizing the best fit model in the Joinpoint Regression Program. The Annual Percent Change (APC) for each segment and the Average Annual Percent Change (AAPC) for the entire study period, with the 95% confidence intervals (CIs) were calculated. APC reflects the average yearly percentage change in rates within a specific time segment, as determined by Joinpoint regression analysis. Whereas, the AAPC summarizes the overall trend across the entire study period by calculating a weighted average of the APCs from each segment. Together, these measures provide a comprehensive framework for quantifying and interpreting long-term changes in mortality. The Monte Carlo permutation method was used for the statistical analysis of the data. A two-sided p-value < 0.05 was considered statistically significant. Values that were found to be statistically significant are marked with an asterisk, ‘*’, in the results.

## Results

### Overall

**Fig. 1:**
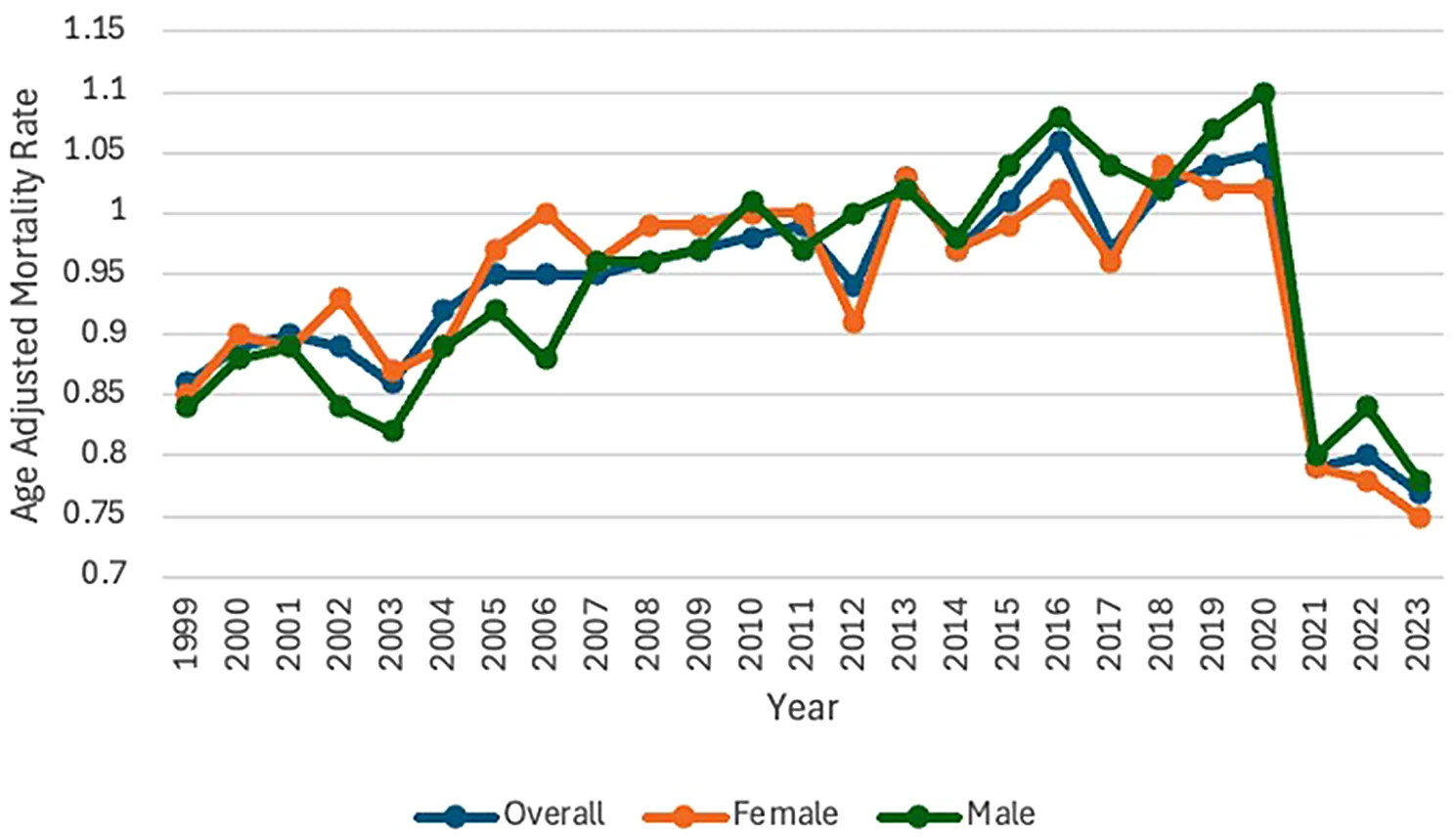
Age adjusted mortality rate in thyroid neoplasm related deaths overall and stratified by sex between 1999 and 2023

Between 1999 and 2023, a total of 53,145 deaths were attributed to malignant neoplasms of the thyroid within this study population, with a significant Joinpoint being observed in 2019. From 1999 to 2019, the APC in AAMR was 0.8981* (95% CI 0.6038 to 1.2327), which then decreased during 2019 to 2023 to -8.1551* (95% CI -12.6712 to -5.2941). Across the full study period, AAMR decreased significantly from 0.86 in 1999 to 0.77 in 2023 with an AAPC of -0.6705* (95% CI -1.082 to -0.3175) (Fig. 1; Supplemental Tables 1–2).

### Sex stratified

During the study period, male and female patients experienced increases in AAMR through 2019–2020 and significant decreases then through 2023. Among men, a significant Joinpoint was detected in 2020. AAMR significantly increased from 1999 to 2020 (APC 1.1478*, 95% CI 0.8274 to 1.5453) and then significantly decreased from 2020 to 2023 (APC − 12.0008*, 95% CI -23.054 to -7.1893), resulting in a net change from 0.84 in 1999 to 0.78 in 2023 (AAPC − 0.5976*, 95% CI -1.3105 to -0.2208). Among women, a significant Joinpoint was detected in 2019. AAMR significantly increased from 1999 to 2019 (APC 0.7045*, 95% CI 0.3486 to 1.1155) and then significantly decreased from 2019 to 2023 (APC − 8.5978*, 95% CI -12.931 to -5.592), yielding a net change from 0.85 (1999) to 0.75 (2023) (AAPC − 0.9092*, 95% CI -1.2852 to -0.5759) (Table 1; Fig. 1; Supplemental Tables 1–2, Supplemental Fig. 1).

**Table 1:** AAMR for Overall and by Sex from 1999–2023

Year	Overall	Female	Male
1999	0.86	0.85	0.84
2000	0.89	0.9	0.88
2001	0.9	0.89	0.89
2002	0.89	0.93	0.84
2003	0.86	0.87	0.82
2004	0.92	0.89	0.89
2005	0.95	0.97	0.92
2006	0.95	1	0.88
2007	0.95	0.96	0.96
2008	0.96	0.99	0.96
2009	0.97	0.99	0.97
2010	0.98	1	1.01
2011	0.99	1	0.97
2012	0.94	0.91	1
2013	1.03	1.03	1.02
2014	0.97	0.97	0.98
2015	1.01	0.99	1.04
2016	1.06	1.02	1.08
2017	0.97	0.96	1.04
2018	1.02	1.04	1.02
2019	1.04	1.02	1.07
2020	1.05	1.02	1.1
2021	0.79	0.79	0.8
2022	0.8	0.78	0.84
2023	0.77	0.75	0.78

### Urbanization (1999–2020)

**Fig. 2:**
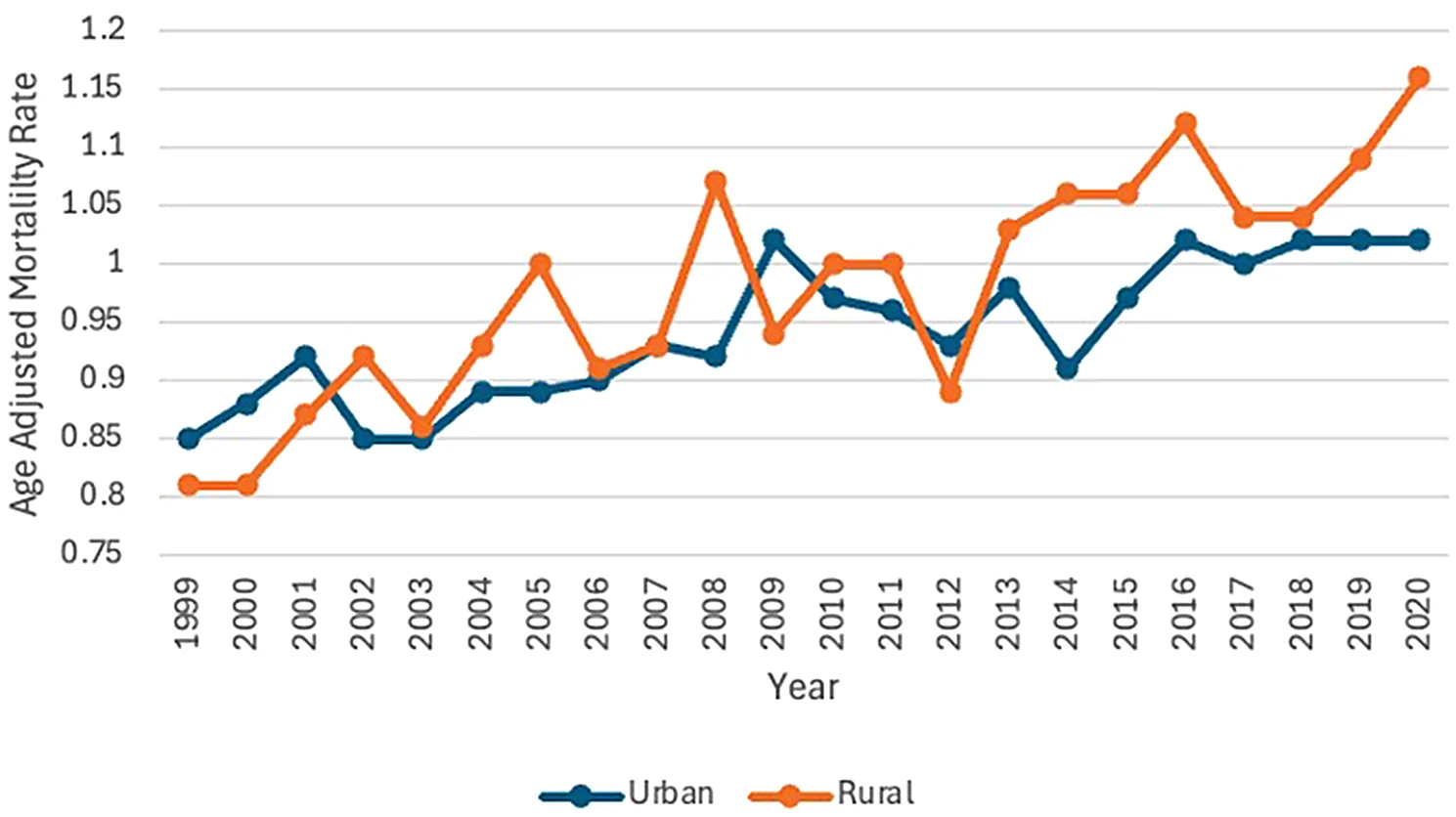
Age adjusted mortality rate in thyroid neoplasm related deaths stratified by urbanization between 1999 and 2020

The CDC Wonder Database did not provide Urbanization data beyond 2020, which is why this subgroup analysis was limited to 1999–2020. We thus cannot report data between 2020 and 2023. Between 1999 and 2020, both rural and urban populations experienced increased malignant thyroid cancer-related mortality. In the rural population, AAMR increased from 0.81 in 1999 to 1.16 in 2020, with a consistent, significant upward trend (AAPC 1.3408*, 95% CI 0.9582 to 1.7976). In the urban population, the AAMR increased from 0.85 in 1999 to 1.02 in 2020 also demonstrating a consistent, significant upward trend (AAPC 0.8426*, 95% CI 0.6096 to 1.1017) (Table 2; Fig. 2; Supplemental Table 1, Supplemental Fig. 2).

**Table 2:** AAMR by Urbanization from 1999–2020

Year	Urban	Rural
1999	0.85	0.81
2000	0.88	0.81
2001	0.92	0.87
2002	0.85	0.92
2003	0.85	0.86
2004	0.89	0.93
2005	0.89	1
2006	0.9	0.91
2007	0.93	0.93
2008	0.92	1.07
2009	1.02	0.94
2010	0.97	1
2011	0.96	1
2012	0.93	0.89
2013	0.98	1.03
2014	0.91	1.06
2015	0.97	1.06
2016	1.02	1.12
2017	1	1.04
2018	1.02	1.04
2019	1.02	1.09
2020	1.02	1.16

### Race

**Fig. 3:**
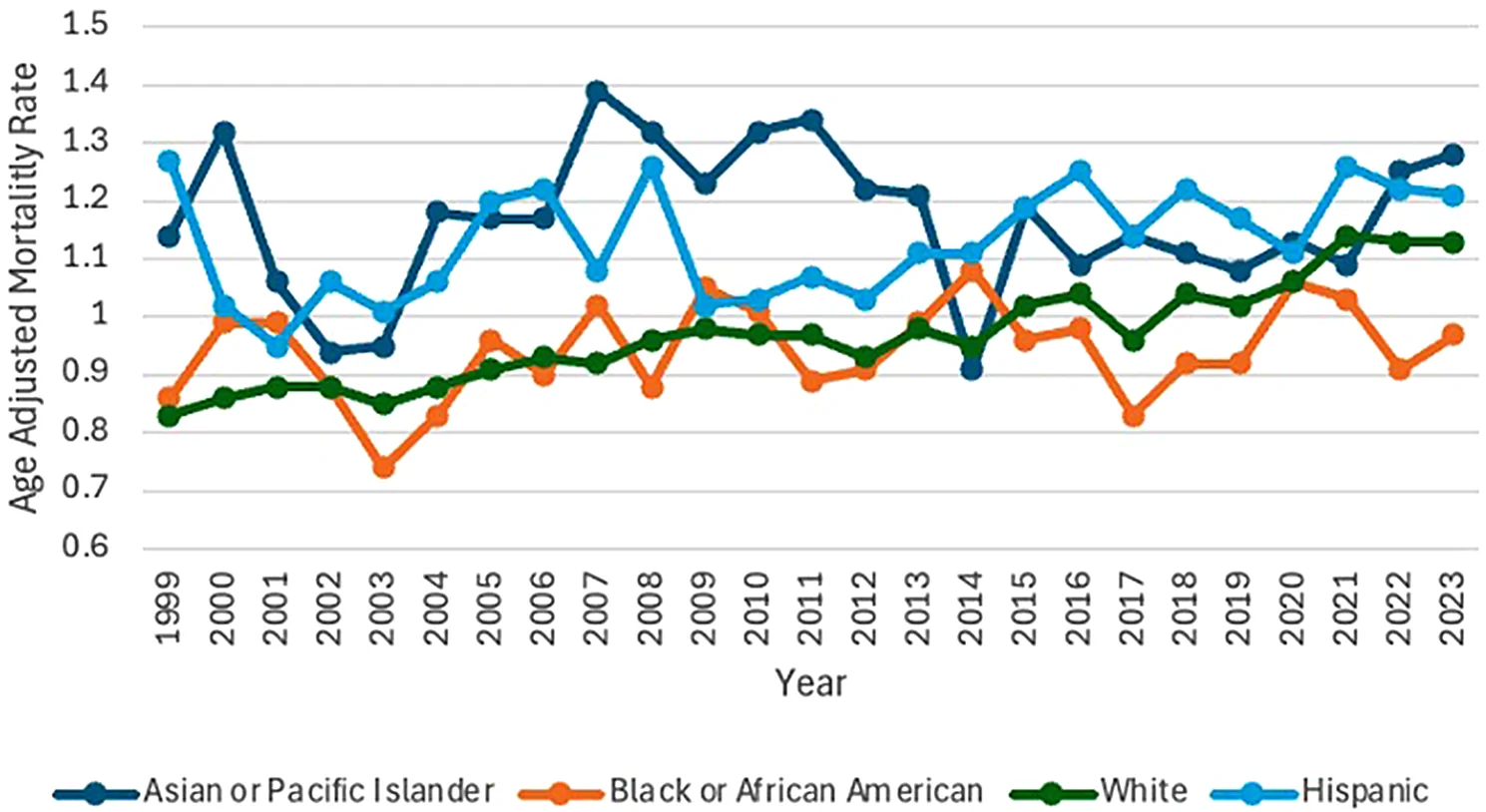
Age adjusted mortality rate in thyroid neoplasm related deaths stratified by race between 1999 and 2023

Throughout the study period, Black or African American patients and White patients had consistently lower thyroid cancer-related mortality than Asian or Pacific Islander and Hispanic patients. Despite these overall mortality trends, Asian or Pacific Islander patients demonstrated relatively stable AAMRs throughout the study period, with no statistically significant change observed (AAPC: -0.0276, 95% CI -0.6655 to 0.8755). All other racial groups experienced an increase in overall AAMR, with White and Hispanic racial subgroups experiencing statistically significant increases (AAPC: 1.1704*, 95% CI 0.9964 to 1.3645, and 0.5675*, 95% CI 0.0863 to 1.2012, respectively). Black or African American patients demonstrated relatively stable AAMRs across the study period. The AAMR was 0.86 in 1999 and 0.97 in 2023, with no statistically significant change observed over time (AAPC: 0.3129, 95% CI, -0.2136 to 0.9438) (Table 3; Fig. 3; Supplemental Table 1).

**Table 3:** AAMR by Race from 1999–2023

Year	Asian or Pacific Islander	Black or African American	White	Hispanic
1999	1.14	0.86	0.83	1.27
2000	1.32	0.99	0.86	1.02
2001	1.06	0.99	0.88	0.95
2002	0.94	0.88	0.88	1.06
2003	0.95	0.74	0.85	1.01
2004	1.18	0.83	0.88	1.06
2005	1.17	0.96	0.91	1.2
2006	1.17	0.9	0.93	1.22
2007	1.39	1.02	0.92	1.08
2008	1.32	0.88	0.96	1.26
2009	1.23	1.05	0.98	1.02
2010	1.32	1.01	0.97	1.03
2011	1.34	0.89	0.97	1.07
2012	1.22	0.91	0.93	1.03
2013	1.21	0.99	0.98	1.11
2014	0.91	1.08	0.95	1.11
2015	1.19	0.96	1.02	1.19
2016	1.09	0.98	1.04	1.25
2017	1.14	0.83	0.96	1.14
2018	1.11	0.92	1.04	1.22
2019	1.08	0.92	1.02	1.17
2020	1.13	1.06	1.06	1.11
2021	1.09	1.03	1.14	1.26
2022	1.25	0.91	1.13	1.22
2023	1.28	0.97	1.13	1.21

### Ten-year age groups

**Fig. 4:**
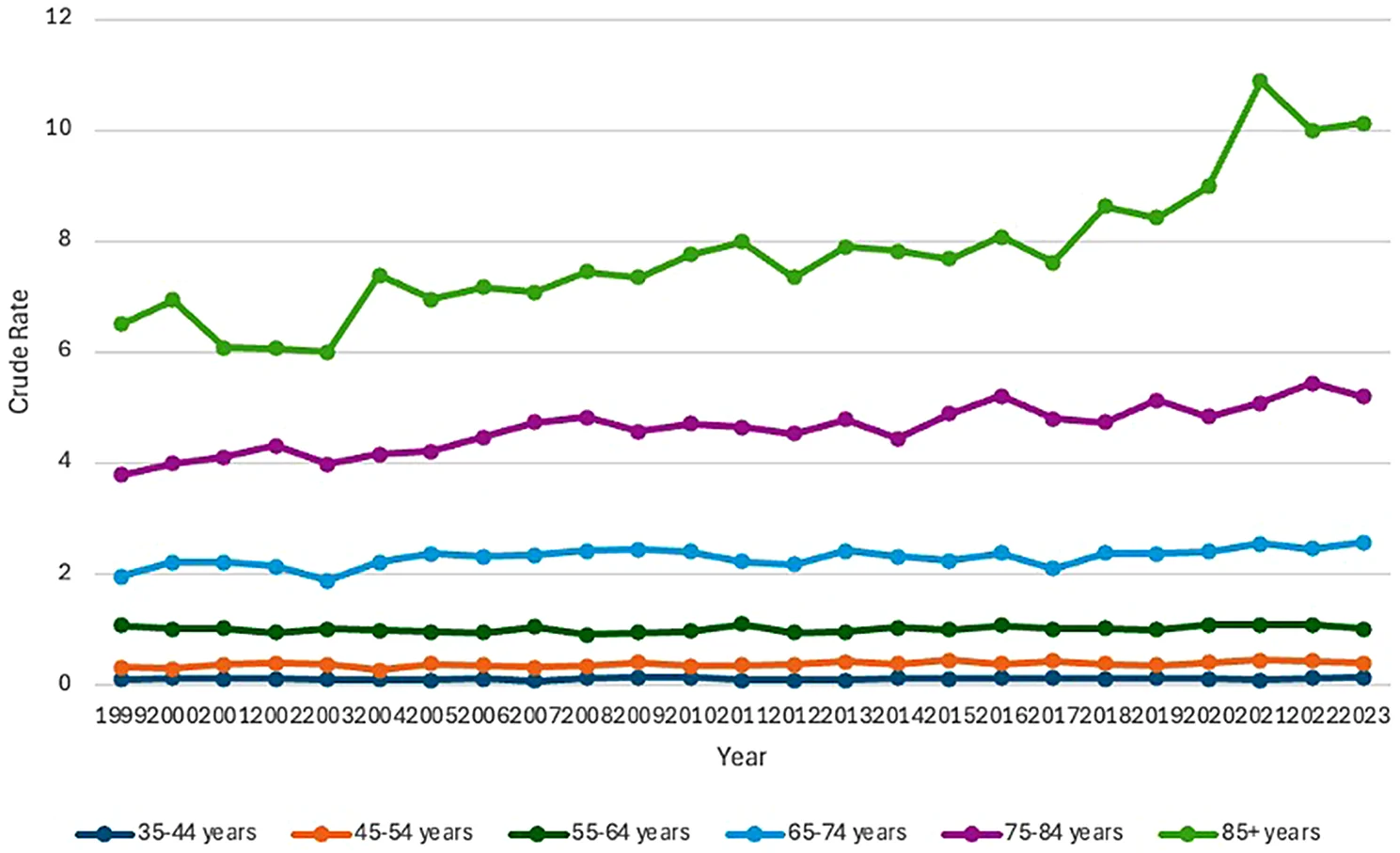
Age adjusted mortality rate in thyroid neoplasm related deaths stratified by 10-year age groups between 1999 and 2023

Age stratified analyses demonstrated increasing AAMR in older cohorts, with the 85 + cohort demonstrating the most pronounced change. Within the 85 + cohort, a significant Joinpoint was identified in 2017. From 1999 to 2017, individuals in this cohort demonstrated relatively stable AAMRs, with no statistically significant change observed over time (APC: 1.3001, 95% CI -1.664 to 1.9416). From 2017 to 2023, however, a significant increase in AAMR among the 85 + age cohort was observed (APC: 4.5147*, 95% CI 2.2188 to 11.4574). Both the 75–84 and 65–74 year old cohorts experienced consistent, significant increases in AAMR across the study period (APC: 1.1162*, 95% CI 0.8472 to 1.4171 and APC: 0.6142*, 95% CI 0.2767 to 1.0176 respectively). Individuals in the 45–54 age cohort also experienced a significant, consistent increase in AAMR across the study period (APC: 1.0019*, 95% CI 0.2659 to 1.8565). Those in the 55–64 and 35–44 age cohorts demonstrated relatively stable AAMRs across the study period, with no statistically significant changes observed (APC: 0.297, 95% CI: -0.0772 to 0.7286, and APC: 0.5488, 95% CI: -0.6127 to 1.779), respectively (Table 4; Fig. 4; Supplemental Tables 1–2, Supplemental Fig. 4).

**Table 4:** AAMR by 10-year Age Groups from 1999–2023

Year	35–44 years	45–54 years	55–64 years	65–74 years	75–84 years	85 + years
1999	0.1	0.31	1.07	1.95	3.79	6.5
2000	0.12	0.29	1.01	2.21	3.99	6.93
2001	0.11	0.37	1.02	2.21	4.1	6.08
2002	0.11	0.39	0.95	2.14	4.31	6.07
2003	0.1	0.37	1.01	1.88	3.98	6
2004	0.1	0.27	0.98	2.21	4.16	7.39
2005	0.09	0.38	0.96	2.37	4.21	6.95
2006	0.11	0.35	0.95	2.31	4.47	7.17
2007	0.07	0.31	1.04	2.34	4.74	7.08
2008	0.13	0.34	0.91	2.41	4.82	7.45
2009	0.14	0.4	0.95	2.44	4.57	7.34
2010	0.14	0.34	0.97	2.4	4.71	7.75
2011	0.08	0.36	1.1	2.23	4.64	7.98
2012	0.09	0.37	0.94	2.17	4.53	7.35
2013	0.08	0.42	0.96	2.42	4.79	7.9
2014	0.12	0.38	1.03	2.31	4.44	7.82
2015	0.11	0.45	0.99	2.24	4.89	7.68
2016	0.13	0.38	1.07	2.38	5.21	8.07
2017	0.13	0.43	1.01	2.1	4.8	7.61
2018	0.11	0.38	1.02	2.38	4.74	8.62
2019	0.13	0.35	0.99	2.36	5.13	8.42
2020	0.11	0.41	1.08	2.4	4.84	8.98
2021	0.09	0.44	1.09	2.55	5.08	10.88
2022	0.12	0.43	1.08	2.45	5.44	9.99
2023	0.14	0.39	1.01	2.57	5.2	10.12

### Region

**Fig. 5:**
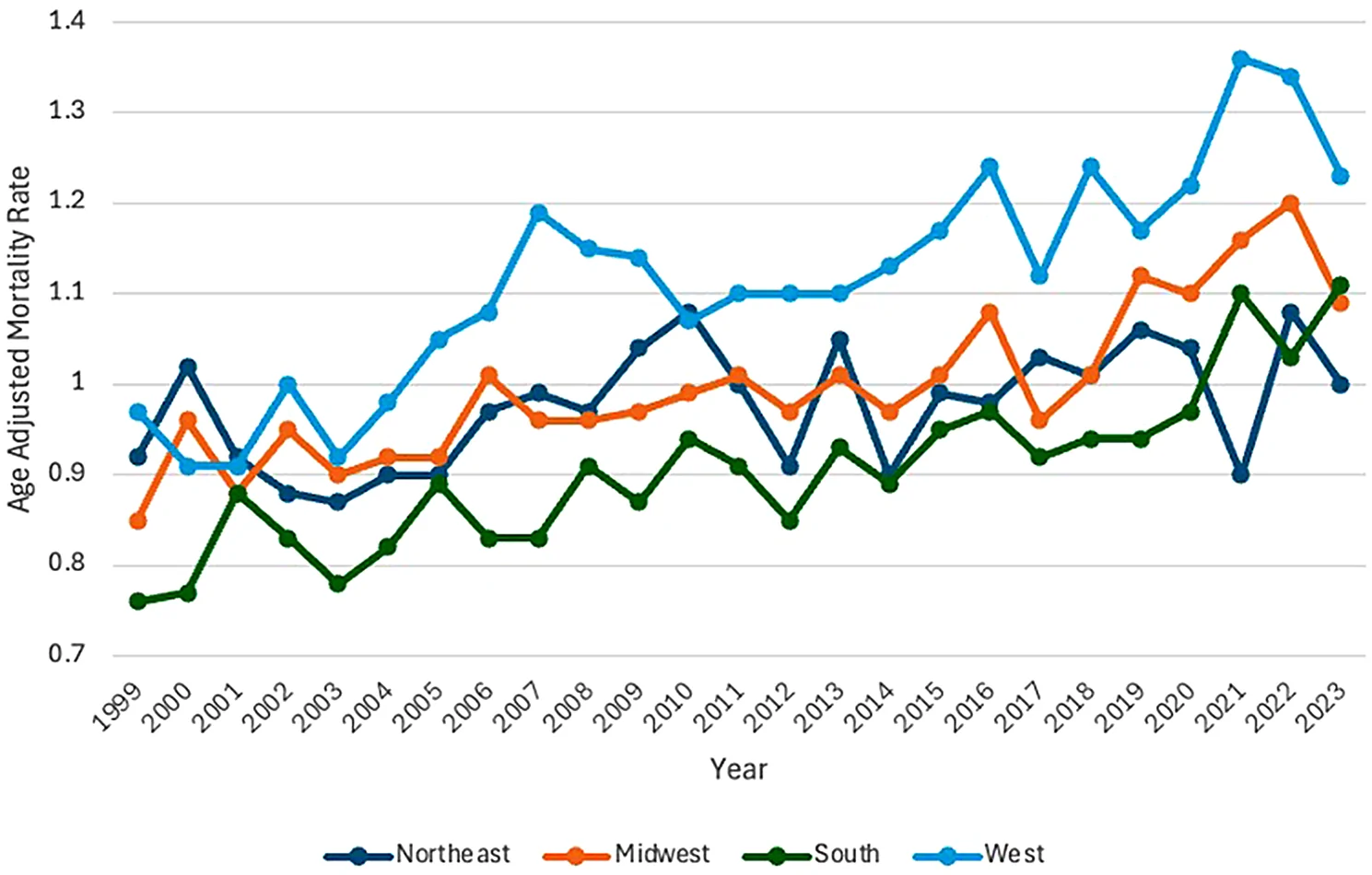
Age adjusted mortality rate in thyroid neoplasm related deaths stratified by region between 1999 and 2023

Malignant thyroid cancer-related mortality demonstrated geographic and temporal variability from 1999 to 2023. In the South region, a significant Joinpoint was identified in 2019. From 1999 to 2019, AAMRs remained relatively stable, with no statistically significant change observed over time (APC: 1.0068, 95% CI: -1.8032 to 5.3145). Between 2019 and 2023, a statistically significant increase in AAMR was observed (APC: 3.5448*, 95% CI: 1.0973 to 8.0848). In the Midwest and West region, patients experienced statistically significant increases in AAMR across the entire study period (APC: 0.9724*, 95% CI: 0.6984 to 1.2838 and APC: 1.2851*, 95% CI: 0.9125 to 1.7099 respectively) (Table 5; Fig. 5; Supplemental Table 2, Supplemental Fig. 5).

**Table 5:** AAMR by Region from 1999–2023

Year	Northeast	Midwest	South	West
1999	0.92	0.85	0.76	0.97
2000	1.02	0.96	0.77	0.91
2001	0.92	0.88	0.88	0.91
2002	0.88	0.95	0.83	1
2003	0.87	0.9	0.78	0.92
2004	0.9	0.92	0.82	0.98
2005	0.9	0.92	0.89	1.05
2006	0.97	1.01	0.83	1.08
2007	0.99	0.96	0.83	1.19
2008	0.97	0.96	0.91	1.15
2009	1.04	0.97	0.87	1.14
2010	1.08	0.99	0.94	1.07
2011	1	1.01	0.91	1.1
2012	0.91	0.97	0.85	1.1
2013	1.05	1.01	0.93	1.1
2014	0.9	0.97	0.89	1.13
2015	0.99	1.01	0.95	1.17
2016	0.98	1.08	0.97	1.24
2017	1.03	0.96	0.92	1.12
2018	1.01	1.01	0.94	1.24
2019	1.06	1.12	0.94	1.17
2020	1.04	1.1	0.97	1.22
2021	0.9	1.16	1.1	1.36
2022	1.08	1.2	1.03	1.34
2023	1	1.09	1.11	1.23

## Discussion

This study aimed to investigate thyroid cancer-related mortality trends across different patient subgroups between 1999 and 2023 utilizing the CDC WONDER Database. This study observed that the AAMR due to thyroid cancer significantly declined from 2019 to 2023 (Supplemental Tables 1 and 2). However, this is at odds with prior literature. Several published studies have demonstrated increasing crude mortality and incidence-based mortality trends in thyroid cancer [16, 23, 24]. While crude or incidence-based mortality rates are rising, it is possible that these rates may overestimate the risk experienced by an elderly population, especially as patient age-based demographics continue to shift [22]. Our expanded study period through 2023 may partially explain differences between our findings and prior analyses. In addition, it is worth noting the increased early detection of thyroid cancer over the years via diagnostic imaging such as ultrasound and fine needle aspiration, CT, and MRI. Collectively, these factors may explain why our study observed a downward trend in AAMR that was not consistently identified in earlier reports.

Both male and female patients experienced an overall increase in thyroid cancer-related mortality between 1999 and 2019 followed by a significant decline in mortality in the latter years of the study (2019–2023). The decline in mortality in the latter years was so profound that the overall AAMR during this study period declined despite an initial period of increased AAMR. These findings should be interpreted cautiously, as the sharp decline in mortality after 2019 may reflect pandemic-related competing non-thyroid cancer mortality, disruptions in death reporting or coding practices, or other COVID-19-related healthcare system effects rather than a true improvement in thyroid cancer outcomes [22–25]. Overall, women experienced a slightly greater decrease in malignant thyroid neoplasm-related mortality than men. This finding aligns with previous studies that demonstrated that the prognosis is worse in men possibly due to older age at diagnosis, advanced staging of tumor, and higher prevalence of tumoral BRAF mutation [26]. The sex-specific differences underscore the need to tailor and advance screening and treatment strategies especially in older men who may be at greater risk for aggressive disease.

Between 1999 and 2020, both rural and urban populations experienced increased AAMR related to malignant neoplasms of the thyroid. While the AAMR for both groups increased, the database excludes the urbanization classification post 2020, during which we observe significant decline in AAMR. During our study period, there was a higher increase in rural mortality with an AAPC of 1.34 compared to the urban AAPC of 0.84. This trend aligns with prior research highlighting rural-urban differences in thyroid cancer care and outcomes. Rural patients are more likely to have limited equitable access to health and educational resources and as a result may face delays in screening, imaging, surveillance, and treatment [27]. In the US, the overall landscape of rural healthcare has several limitations. For instance, shortages in providers including specialists, fewer hospitals and surgery centers, under-resourced facilities, longer travel distances to health care facilities make health care more difficult [28]. A recent nationwide analysis found that rural counties have virtually no oncologists compared to urban areas (median 0.0 vs. 6.0 per 100,000 population), and this lower specialist density was associated with higher cancer mortality, highlighting structural barriers to timely diagnosis and treatment in rural populations [29]. These findings suggest an urgent need for targeted interventions in the rural areas to improve access to education, diagnostic services, endocrine surgical care, and longitudinal follow up.

White and Hispanic patients experienced statistically significant increases in AAMR, whereas Black or African American and Asian or Pacific Islander patients demonstrated a relatively stable mortality trends. Although the Asian or Pacific Islander subgroup demonstrated relatively stable mortality trend, this population consistently maintained a higher AAMR than the other racial subgroups during the same time period. The National Cancer Institute’s SEER 12 also recognized the increasing thyroid cancer-related mortality amongst these racial subgroups [2, 23, 24, 30]. One study also identified higher thyroid cancer mortality among Asian patients, potentially related to differences in stage at presentation and tumor characteristics [30]. Studies have also hypothesized that the disproportionate mortality rates amongst these racial subgroups root from their socioeconomic differences, which was based on findings of higher mortality rates amongst Black or African Americans compared to White people [30]. However, this does not corroborate our results, especially based on the mortality rates observed for these two populations after 2010 (Supplemental Fig. 3). There is no clear explanation for observed mortality trends amongst racial subgroups, but these findings underline not only the importance of access to healthcare but also education on risk assessment and patient safety in healthcare. Although our analysis did not account for histologic subtype, stage at diagnosis, or treatment patterns, existing literature demonstrates differences in these factors across racial and ethnic groups. Prior research indicates that the distribution of thyroid cancer histologic subtypes differs across racial and ethnic groups, which likely contributes to observed mortality differences. Non-Hispanic Black patients were more likely to present with aggressive subtypes such as medullary thyroid cancer and follicular thyroid cancer and were less likely to receive the recommended surgical resection, while Hispanic patients more often presented with advanced-stage tumors, including follicular thyroid cancer and anaplastic thyroid cancer, and had lower rates of radiotherapy. In contrast, Non-Hispanic White patients were more likely to present with papillary thyroid cancer, the most common and least aggressive subtype, which is associated with favorable prognosis. These variations in histologic subtype, combined with differences in stage at diagnosis and treatment access, likely mediate a substantial portion of the disparities in age-adjusted mortality rates observed among racial and ethnic groups [31].

The AAMR between 1999 and 2023 followed an overall increasing trend in the 10-year age cohorts, with the highest crude rate of malignant thyroid cancer-related deaths observed in the oldest age group of 85 + years, and progressively lower crude rates in younger cohorts. These findings align with previous literature that states that older age groups exhibit a higher incidence and risk of malignant thyroid cancers, cumulative lifetime exposures, age-related biological vulnerability, and increasing longevity within the population [32]. The disproportionate mortality burden in older populations may indicate that the improvements in survival rate have not been equally distributed. Broader demographic shifts resulting in a growing very-elderly population may partially explain the steeper rise in mortality observed after 2017, as more individuals are living to ages at which both cancer risk and competing health conditions increase. This may also suggest the limitations in aggressive treatment options, underutilization of care due to accumulation of comorbidities, increased frailty, and higher competing mortality risks, which can limit eligibility for surgery, radioactive iodine, or other definitive therapies in older patients. Additionally, changing clinical thresholds for surgical resection and adjuvant treatment in frail or medically complex patients may contribute to higher observed mortality in the oldest age groups, even when age-adjusted rates are considered [33–34]. Taken together, these factors highlight that age-related increases in thyroid cancer mortality likely reflect a complex interplay between biology, treatment feasibility, and evolving population demographics rather than age alone.

Throughout the study period, individuals living outside the Northeast experienced significant increases in AAMR. Obesity, a known risk factor for thyroid cancer [11–12], is more prevalent in the South and Midwest regions of the United States [35]. Udelsman and Zhang also noted that the Northeast has the highest concentration of specialized care for thyroid cancer while the South has the lowest concentration. Being in an area with more specialized care increases cancer detection and improves long-term outcomes in the region [36]. In addition to obesity prevalence and access to specialized care, prior studies suggest that regional variation in thyroid cancer outcomes may reflect differences in specialist availability and treatment patterns across the United States. Analyses using data from the Surveillance, Epidemiology, and End Results Program have demonstrated that patients in the Western United States are more likely to present with regional or distant metastatic thyroid cancer compared with other regions, indicating geographic differences in stage at diagnosis that may contribute to mortality trends [37]. More recent work has shown that higher county-level endocrinologist density is independently associated with improved thyroid cancer–specific survival, underscoring the importance of specialist availability in optimizing outcomes. The Northeast, which has a higher concentration of endocrinologists and high-volume thyroid surgeons, may therefore benefit from more timely diagnosis, referral, and guideline-concordant management. In contrast, regional differences in thyroidectomy volume patterns across the United States have also been described, with variation in access to high-volume thyroid surgical care between regions [38]. Together, these regional differences in disease presentation, endocrinology workforce density, and surgical expertise provide a plausible explanation for the relative stability of mortality in the Northeast compared with the increasing age-adjusted mortality rates observed in other regions.

### Limitations

Although this study followed sound research design, CDC WONDER is a publicly available and collected database, which does pose some limitations on gathered results. First, the database compiles data from death certificates of every recorded mortality in the United States. This indicates that if a death is not recorded, it is not included in the database. Authors utilized comprehensive ICD-10 codes to ensure all recorded deaths related to thyroid cancer were gathered, but this study cannot account for those that were not recorded. Second, multiple causes of death can be listed for each patient. This makes it impossible to determine the extent of the impact that thyroid cancer had on an individual’s mortality. While this limitation cannot be completely negated, the use of comprehensive ICD-10 codes ensures that thyroid cancer played a role in every death included in this study. Social determinants of health are also not accounted for in the CDC WONDER database, which could impact mortality data and results.

Another limitation of our study is that this study does not stratify analyses by histopathological subtypes. Thyroid cancer subtypes include papillary, follicular, medullary, and anaplastic. Each subtype exhibits distinct incidence patterns across age, sex, and race/ethnicity, as well as differing biological behavior and aggressiveness [5]. For instance, papillary thyroid cancer is the most common and generally indolent, while anaplastic thyroid cancer is rare but highly aggressive, often presenting later in life with poor prognosis. Aggregating all subtypes in the analysis using CDC WONDER data from 1999 to 2023 may obscure these subtype-specific mortality trends, particularly for rarer or more aggressive tumors. This heterogeneity could lead to underestimation or overestimation of mortality rates for specific subgroups and may limit the interpretability of our overall findings. Future studies leveraging datasets with detailed histopathological coding should perform subtype-specific analyses to better delineate mortality patterns and inform targeted clinical and public health strategies.

Finally, this study focused on the time-period of 1999 to 2023, to include all verified data that is available up to the present. This indicates that the COVID-19 pandemic could have impacted results from 2020 onward. Due to factors such as lockdown and isolation measures, fear of contracting the virus, or limited access to in-person healthcare, the pandemic may have contributed to an increase in thyroid cancer mortality due to reduced access to surgical and medical management. However, several studies did not observe this trend and attributed this to the variable levels of burden with hospital staffing and resources as well as the prioritization of oncologic care during the pandemic [26]. On the other hand, Hassan et al. compared pre-pandemic and post-pandemic histopathologic features of papillary thyroid cancer patients in Abu Dhabi, and the study observed tumors decreasing in median size but increasingly aggressive and invasive [39]. Since Abu Dhabi facilities did not have any restrictions during the pandemic, Hassan et al. supported theories of a multifactorial etiology rooting from causes like psychological stress or inflammatory stress of COVID-19 infections resulting in further immune dysregulation [39]. In theory, this trend should have led to an increase in thyroid cancer mortality rate following the pandemic. Though several studies did not observe those expected findings, the above hypotheses demonstrate the many possibilities in which the COVID-19 pandemic could have influenced reported thyroid mortality rates. This study does not isolate the pandemic period to determine the specific impact that COVID-19 may have had, but it works to include as much data as possible to provide overall trends in thyroid cancer mortality to help inform future care.

## Conclusion

Between 1999 and 2023, the US saw an overall decrease in mortality attributed to malignant thyroid neoplasms. Despite this overall decrease, variations between groups based on race, and geographic region persist. These findings highlight persistent disparities in thyroid cancer mortality across sex, age, race, and geographic regions, despite overall declines in AAMR. Targeted interventions are warranted to address these inequities. Specifically, healthcare policy should prioritize improving access to thyroid cancer screening, diagnostic imaging, and endocrine surgical care in rural and underserved regions. Tailored public health education campaigns can raise awareness of thyroid cancer risk factors among high-risk populations, including older adults and racial subgroups experiencing disproportionate mortality. Additionally, strategies to ensure equitable allocation of healthcare resources, such as specialist availability and early referral pathways, may help reduce regional and demographic disparities in outcomes. Future interventions should be guided by these trends to optimize early detection and improve survival across all populations.

## Supplementary Information

Below is the link to the electronic supplementary material.

Supplementary Material 1

## Data Availability

The datasets analyzed during the current study are publicly available through the CDC WONDER online database (https://wonder.cdc.gov/mcd.html).

## Abbreviations

AAMR: Age–Adjusted Mortality Rate
AAPC: Average Annual Percent Change
APC: Annual Percent Change
CDC WONDER: Centers for Disease Control and Prevention Wide–ranging Online Data for Epidemiologic Research
CI: Confidence Interval
DTC: Differentiated Thyroid Cancer
ICD: 10–International Classification of Diseases, 10th Revision
IRB: Institutional Review Board
RAI: Radioactive Iodine
SEER: Surveillance, Epidemiology, and End Results
