# Supplementary material for: Trends and disparities in thyroid cancer related mortality from 1999 to 2023: a CDC WONDER database analysis
Source: BMC Endocr Disord. 2026 Jul 7;26:258. doi: 10.1186/s12902-026-02402-y (PMC13548645; doi:10.1186/s12902-026-02402-y)
Supplement: Supplementary file 1 [file 12902_2026_2402_MOESM1_ESM.docx]

**Supplemental Table 1**: Annual Average Percent Change (AAPC) in Malignant Thyroid Neoplasms Related Deaths by Patient Subgroup

| Annual Average Percent Change (AAPC) | | | | | |
| --- | --- | --- | --- | --- | --- |
| **Cohort** | **Years** | **AAPC** | **Lower CI** | **Upper CI** | **P-Value** |
| Overall | 1999-2023 | **-0.6705*** | -1.082 | -0.3175 | **0.0012** |
|  |  |  |  |  |  |
| Female | 1999-2023 | **-0.9092*** | -1.2852 | -0.5759 | **< 0.000001** |
| Male | 1999-2023 | **-0.5976*** | -1.3105 | -0.2208 | **0.0016** |
|  |  |  |  |  |  |
| Asian or Pacific Islander | 1999-2023 | -0.0276 | -0.6655 | 0.8755 | 0.842232 |
| Black or African American | 1999-2023 | 0.3129 | -0.2136 | 0.9438 | 0.226755 |
| White | 1999-2023 | **1.1704*** | 0.9964 | 1.3645 | **< 0.000001** |
| Hispanic | 1999-2023 | **0.5675*** | 0.0863 | 1.2012 | **0.028794** |
|  |  |  |  |  |  |
| Northeast | 1999-2023 | 0.3884 | -0.0484 | 0.8686 | 0.078784 |
| Midwest | 1999-2023 | **0.9724*** | 0.6984 | 1.2838 | **< 0.000001** |
| South | 1999-2023 | **1.4254*** | 0.9467 | 1.8508 | **< 0.000001** |
| West | 1999-2023 | **1.2851*** | 0.9125 | 1.7099 | **< 0.000001** |
|  |  |  |  |  |  |
| Urban | 1999-2020 | **0.8426*** | 0.6096 | 1.1017 | **< 0.000001** |
| Rural | 1999-2020 | **1.3408*** | 0.9582 | 1.7976 | **< 0.000001** |
|  |  |  |  |  |  |
| 35-44 years | 1999-2023 | 0.5488 | -0.6127 | 1.779 | 0.371126 |
| 45-54 years | 1999-2023 | **1.0019*** | 0.2659 | 1.8565 | **0.011998** |
| 55-64 years | 1999-2023 | 0.297 | -0.0772 | 0.7286 | 0.109578 |
| 65-74 years | 1999-2023 | **0.6142*** | 0.2767 | 1.0176 | **0.0004** |
| 75-84 years | 1999-2023 | **1.1162*** | 0.8472 | 1.4171 | **< 0.000001** |
| 85+ years | 1999-2023 | **2.0944*** | 1.4968 | 2.6141 | **< 0.000001** |

**Supplemental Table 2**: Annual Percent Change (APC) in Malignant Thyroid Neoplasms Related Deaths by Patient Subgroup

| Annual Percent Change (APC) | | | | | |
| --- | --- | --- | --- | --- | --- |
| **Cohort** | **Years** | **APC** | **Lower CI** | **Upper CI** | **P-Value** |
| Overall | 1999-2019 | **0.8981*** | 0.6038 | 1.2327 | **< 0.000001** |
| Overall | 2019-2023 | **-8.1551*** | -12.6712 | -5.2941 | **< 0.000001** |
|  |  |  |  |  |  |
| Female | 1999-2019 | **0.7045*** | 0.3486 | 1.1155 | **< 0.000001** |
| Female | 2019-2023 | **-8.5978*** | -12.931 | -5.592 | **< 0.000001** |
|  |  |  |  |  |  |
| Male | 1999-2020 | **1.1478*** | 0.8274 | 1.5453 | **< 0.000001** |
| Male | 2020-2023 | **-12.0008*** | -23.054 | -7.1893 | **< 0.000001** |
|  |  |  |  |  |  |
| Asian or Pacific Islander | 1999-2023 | -0.0276 | -0.6655 | 0.8755 | 0.842232 |
| Black or African American | 1999-2023 | 0.3129 | -0.2136 | 0.9438 | 0.226755 |
| White | 1999-2023 | **1.1704*** | 0.9964 | 1.3645 | **< 0.000001** |
| Hispanic | 1999-2023 | **0.5675*** | 0.0863 | 1.2012 | **0.028794** |
|  |  |  |  |  |  |
| Northeast | 1999-2023 | 0.3884 | -0.0484 | 0.8686 | 0.078784 |
| Midwest | 1999-2023 | **0.9724*** | 0.6984 | 1.2838 | **< 0.000001** |
| South | 1999-2019 | 1.0068 | -1.8032 | 5.3145 | 0.158768 |
| South | 2019-2023 | **3.5448*** | 1.0973 | 8.0848 | **0.026795** |
| West | 1999-2023 | **1.2851*** | 0.9125 | 1.7099 | **< 0.000001** |
|  |  |  |  |  |  |
| Urban | 1999-2020 | **0.8426*** | 0.6096 | 1.1017 | **< 0.000001** |
| Rural | 1999-2020 | **1.3408*** | 0.9582 | 1.7976 | **< 0.000001** |
|  |  |  |  |  |  |
| 35-44 years | 1999-2023 | 0.5488 | -0.6127 | 1.779 | 0.371126 |
| 45-54 years | 1999-2023 | **1.0019*** | 0.2659 | 1.8565 | **0.011998** |
| 55-64 years | 1999-2023 | 0.297 | -0.0772 | 0.7286 | 0.109578 |
| 65-74 years | 1999-2023 | **0.6142*** | 0.2767 | 1.0176 | **0.0004** |
| 75-84 years | 1999-2023 | **1.1162*** | 0.8472 | 1.4171 | **< 0.000001** |
| 85+ years | 1999-2017 | 1.3001 | -1.6646 | 1.9416 | 0.126375 |
| 85+ years | 2017-2023 | **4.5147*** | 2.2188 | 11.4574 | **0.003999** |

***Supplemental Figure 1***: Multiple Joinpoint Model Malignant Thyroid Neoplasm Related Deaths Overall and Stratified by Sex between 1999 and 2023.

**Supplemental Figure 2**: Multiple Joinpoint Model Malignant Thyroid Neoplasm Related Deaths Stratified by Urbanization between 1999 and 2020.

**Supplemental Figure 3**: Multiple Joinpoint Model Malignant Thyroid Neoplasm Related Deaths Stratified by Race between 1999 and 2023.

**Supplemental Figure 4**: Multiple Joinpoint Model Malignant Thyroid Neoplasm Related Deaths Stratified by 10-year age groups between 1999 and 2023.

**Supplemental Figure 5**: Multiple Joinpoint Model Malignant Thyroid Neoplasm Related Deaths Stratified by Region between 1999 and 2023.

**Supplemental Table 3a**: Population and Death Count of the entire sample

| **Year** | **Deaths** | **Population** |
| --- | --- | --- |
| 1999 | 1518 | 180408769 |
| 2000 | 1612 | 181984640 |
| 2001 | 1651 | 184305128 |
| 2002 | 1680 | 186208028 |
| 2003 | 1623 | 188090429 |
| 2004 | 1740 | 190205384 |
| 2005 | 1835 | 192551384 |
| 2006 | 1896 | 195019359 |
| 2007 | 1962 | 197403777 |
| 2008 | 2045 | 199795090 |
| 2009 | 2095 | 202107016 |
| 2010 | 2144 | 203891983 |
| 2011 | 2201 | 206592936 |
| 2012 | 2130 | 208826037 |
| 2013 | 2349 | 211085314 |
| 2014 | 2340 | 213809280 |
| 2015 | 2448 | 216553817 |
| 2016 | 2611 | 218641417 |
| 2017 | 2493 | 221447331 |
| 2018 | 2680 | 223311190 |
| 2019 | 2757 | 224981167 |
| 2020 | 2870 | 226635013 |
| 2021 | 2105 | 228238412 |
| 2022 | 2202 | 229508599 |
| 2023 | 2147 | 231529762 |

**Supplemental Table 3b**: Population and Death Count by Sex from 1999-2023

| **Sex** | **Year** | **Deaths** | **Population** |  | **Sex** | **Year** | **Deaths** | **Population** |
| --- | --- | --- | --- | --- | --- | --- | --- | --- |
| Female | 1999 | 887 | 94123092 |  | Male | 1999 | 631 | 86285677 |
| Female | 2000 | 955 | 94864102 |  | Male | 2000 | 657 | 87120538 |
| Female | 2001 | 960 | 95984408 |  | Male | 2001 | 691 | 88320720 |
| Female | 2002 | 1001 | 96927703 |  | Male | 2002 | 679 | 89280325 |
| Female | 2003 | 953 | 97893297 |  | Male | 2003 | 670 | 90197132 |
| Female | 2004 | 1008 | 98921524 |  | Male | 2004 | 732 | 91283860 |
| Female | 2005 | 1081 | 100097839 |  | Male | 2005 | 754 | 92453545 |
| Female | 2006 | 1134 | 101328442 |  | Male | 2006 | 762 | 93690917 |
| Female | 2007 | 1125 | 102513830 |  | Male | 2007 | 837 | 94889947 |
| Female | 2008 | 1192 | 103688156 |  | Male | 2008 | 853 | 96106934 |
| Female | 2009 | 1201 | 104834186 |  | Male | 2009 | 894 | 97272830 |
| Female | 2010 | 1220 | 105717426 |  | Male | 2010 | 924 | 98174557 |
| Female | 2011 | 1249 | 107004480 |  | Male | 2011 | 952 | 99588456 |
| Female | 2012 | 1158 | 108088829 |  | Male | 2012 | 972 | 100737208 |
| Female | 2013 | 1317 | 109172782 |  | Male | 2013 | 1032 | 101912532 |
| Female | 2014 | 1316 | 110581619 |  | Male | 2014 | 1024 | 103227661 |
| Female | 2015 | 1339 | 111947362 |  | Male | 2015 | 1109 | 104606455 |
| Female | 2016 | 1442 | 112990913 |  | Male | 2016 | 1169 | 105650504 |
| Female | 2017 | 1335 | 114358026 |  | Male | 2017 | 1158 | 107089305 |
| Female | 2018 | 1503 | 115265998 |  | Male | 2018 | 1177 | 108045192 |
| Female | 2019 | 1505 | 116084432 |  | Male | 2019 | 1252 | 108896735 |
| Female | 2020 | 1557 | 116909132 |  | Male | 2020 | 1313 | 109725881 |
| Female | 2021 | 1154 | 116838617 |  | Male | 2021 | 951 | 111399795 |
| Female | 2022 | 1181 | 117317971 |  | Male | 2022 | 1021 | 112190628 |
| Female | 2023 | 1142 | 118635845 |  | Male | 2023 | 1005 | 112893917 |

**Supplemental Table 3c**: Population and Death Count by Urbanization from 1999-2020

| Urban/Rural | Year | Deaths | Population |
| --- | --- | --- | --- |
| Urban | 1999 | 782 | 96087132 |
| Urban | 2000 | 838 | 97067584 |
| Urban | 2001 | 893 | 98567292 |
| Urban | 2002 | 862 | 99841258 |
| Urban | 2003 | 857 | 101111103 |
| Urban | 2004 | 922 | 102566358 |
| Urban | 2005 | 939 | 104142434 |
| Urban | 2006 | 1000 | 105855168 |
| Urban | 2007 | 1029 | 107383222 |
| Urban | 2008 | 1070 | 108858478 |
| Urban | 2009 | 1154 | 110213357 |
| Urban | 2010 | 1155 | 111277952 |
| Urban | 2011 | 1188 | 112744933 |
| Urban | 2012 | 1174 | 113971649 |
| Urban | 2013 | 1270 | 115107744 |
| Urban | 2014 | 1223 | 116598502 |
| Urban | 2015 | 1299 | 118126813 |
| Urban | 2016 | 1413 | 119455722 |
| Urban | 2017 | 1388 | 121017197 |
| Urban | 2018 | 1500 | 122301495 |
| Urban | 2019 | 1531 | 123459254 |
| Urban | 2020 | 1577 | 124676398 |
|  | | | |
| Rural | 1999 | 266 | 29163427 |
| Rural | 2000 | 278 | 29325941 |
| Rural | 2001 | 291 | 29408870 |
| Rural | 2002 | 317 | 29548977 |
| Rural | 2003 | 296 | 29722714 |
| Rural | 2004 | 323 | 29933287 |
| Rural | 2005 | 352 | 30179558 |
| Rural | 2006 | 335 | 30495970 |
| Rural | 2007 | 346 | 30752891 |
| Rural | 2008 | 384 | 30969063 |
| Rural | 2009 | 354 | 31141442 |
| Rural | 2010 | 375 | 31300878 |
| Rural | 2011 | 384 | 31388404 |
| Rural | 2012 | 351 | 31402361 |
| Rural | 2013 | 407 | 31450865 |
| Rural | 2014 | 420 | 31505264 |
| Rural | 2015 | 432 | 31594511 |
| Rural | 2016 | 454 | 31678227 |
| Rural | 2017 | 429 | 31772269 |
| Rural | 2018 | 442 | 31896166 |
| Rural | 2019 | 471 | 31974679 |
| Rural | 2020 | 520 | 32028405 |

**Supplemental Table 3d**: Population and Death Count by Race from 1999-2023

| Race | Year | Deaths | Population |
| --- | --- | --- | --- |
| American Indian or Alaska Native | 2007 | 10 | 1434577 |
| American Indian or Alaska Native | 2011 | 12 | 1533376 |
| American Indian or Alaska Native | 2013 | 20 | 1581241 |
| American Indian or Alaska Native | 2014 | 11 | 1608821 |
| American Indian or Alaska Native | 2015 | 11 | 1637629 |
| American Indian or Alaska Native | 2016 | 11 | 1667114 |
| American Indian or Alaska Native | 2017 | 16 | 1695440 |
| American Indian or Alaska Native | 2018 | 14 | 1722458 |
| American Indian or Alaska Native | 2019 | 16 | 1746677 |
| American Indian or Alaska Native | 2020 | 14 | 1771292 |
| American Indian or Alaska Native | 2021 | 19 | 1604632 |
| American Indian or Alaska Native | 2022 | 22 | 1599267 |
| American Indian or Alaska Native | 2023 | 13 | 1625106 |
|  | | | |
| Asian or Pacific Islander | 1999 | 47 | 6917321 |
| Asian or Pacific Islander | 2000 | 61 | 7210256 |
| Asian or Pacific Islander | 2001 | 49 | 7651274 |
| Asian or Pacific Islander | 2002 | 53 | 7999855 |
| Asian or Pacific Islander | 2003 | 55 | 8336941 |
| Asian or Pacific Islander | 2004 | 66 | 8679634 |
| Asian or Pacific Islander | 2005 | 72 | 9041913 |
| Asian or Pacific Islander | 2006 | 77 | 9409587 |
| Asian or Pacific Islander | 2007 | 100 | 9760888 |
| Asian or Pacific Islander | 2008 | 94 | 10102040 |
| Asian or Pacific Islander | 2009 | 96 | 10429408 |
| Asian or Pacific Islander | 2010 | 103 | 10670562 |
| Asian or Pacific Islander | 2011 | 115 | 11055808 |
| Asian or Pacific Islander | 2012 | 111 | 11510641 |
| Asian or Pacific Islander | 2013 | 111 | 11931284 |
| Asian or Pacific Islander | 2014 | 95 | 12503895 |
| Asian or Pacific Islander | 2015 | 130 | 13054155 |
| Asian or Pacific Islander | 2016 | 125 | 13344756 |
| Asian or Pacific Islander | 2017 | 138 | 13938546 |
| Asian or Pacific Islander | 2018 | 138 | 14258765 |
| Asian or Pacific Islander | 2019 | 140 | 14536839 |
| Asian or Pacific Islander | 2020 | 163 | 14798344 |
| Asian or Pacific Islander | 2021 | 148 | 14425606 |
| Asian or Pacific Islander | 2022 | 178 | 14877327 |
| Asian or Pacific Islander | 2023 | 191 | 15269889 |
|  | | | |
| Black or African American | 1999 | 132 | 19808077 |
| Black or African American | 2000 | 155 | 20058273 |
| Black or African American | 2001 | 156 | 20404945 |
| Black or African American | 2002 | 138 | 20695836 |
| Black or African American | 2003 | 115 | 20982115 |
| Black or African American | 2004 | 139 | 21327519 |
| Black or African American | 2005 | 167 | 21703691 |
| Black or African American | 2006 | 158 | 22092072 |
| Black or African American | 2007 | 178 | 22474128 |
| Black or African American | 2008 | 162 | 22857307 |
| Black or African American | 2009 | 199 | 23236715 |
| Black or African American | 2010 | 198 | 23537629 |
| Black or African American | 2011 | 182 | 23958352 |
| Black or African American | 2012 | 191 | 24345607 |
| Black or African American | 2013 | 220 | 24743381 |
| Black or African American | 2014 | 235 | 25244585 |
| Black or African American | 2015 | 220 | 25752287 |
| Black or African American | 2016 | 232 | 26212105 |
| Black or African American | 2017 | 204 | 26762620 |
| Black or African American | 2018 | 238 | 27178229 |
| Black or African American | 2019 | 246 | 27592123 |
| Black or African American | 2020 | 281 | 27969928 |
| Black or African American | 2021 | 268 | 27538737 |
| Black or African American | 2022 | 245 | 27685911 |
| Black or African American | 2023 | 258 | 28042483 |
|  | | | |
| White | 1999 | 1217 | 134935890 |
| White | 2000 | 1288 | 135202971 |
| White | 2001 | 1338 | 135651679 |
| White | 2002 | 1357 | 136026843 |
| White | 2003 | 1318 | 136415868 |
| White | 2004 | 1390 | 136956601 |
| White | 2005 | 1440 | 137614452 |
| White | 2006 | 1491 | 138363022 |
| White | 2007 | 1523 | 139060265 |
| White | 2008 | 1591 | 139772453 |
| White | 2009 | 1620 | 140451863 |
| White | 2010 | 1664 | 140987505 |
| White | 2011 | 1696 | 141789725 |
| White | 2012 | 1632 | 142424668 |
| White | 2013 | 1784 | 143045234 |
| White | 2014 | 1767 | 143642265 |
| White | 2015 | 1830 | 144347874 |
| White | 2016 | 1967 | 144979180 |
| White | 2017 | 1856 | 145456222 |
| White | 2018 | 1988 | 145801376 |
| White | 2019 | 2049 | 146079678 |
| White | 2020 | 2110 | 146337256 |
| White | 2021 | 2226 | 144655323 |
| White | 2022 | 2314 | 144499701 |
| White | 2023 | 2283 | 144415042 |

**Supplemental Table 3e**: Population and Death Count by Ethnicity from 1999-2023

| Ethnicity | Year | Deaths | Population |
| --- | --- | --- | --- |
| Hispanic | 1999 | 113 | 17503631 |
| Hispanic | 2000 | 100 | 18219679 |
| Hispanic | 2001 | 100 | 19290018 |
| Hispanic | 2002 | 119 | 20159630 |
| Hispanic | 2003 | 121 | 21011656 |
| Hispanic | 2004 | 134 | 21877214 |
| Hispanic | 2005 | 149 | 22804023 |
| Hispanic | 2006 | 162 | 23743864 |
| Hispanic | 2007 | 149 | 24673919 |
| Hispanic | 2008 | 187 | 25602850 |
| Hispanic | 2009 | 164 | 26504021 |
| Hispanic | 2010 | 171 | 27192663 |
| Hispanic | 2011 | 192 | 28255675 |
| Hispanic | 2012 | 187 | 28988437 |
| Hispanic | 2013 | 211 | 29784174 |
| Hispanic | 2014 | 227 | 30809714 |
| Hispanic | 2015 | 254 | 31761872 |
| Hispanic | 2016 | 274 | 32438262 |
| Hispanic | 2017 | 272 | 33594503 |
| Hispanic | 2018 | 299 | 34350362 |
| Hispanic | 2019 | 298 | 35025850 |
| Hispanic | 2020 | 294 | 35758193 |
| Hispanic | 2021 | 345 | 36687942 |
| Hispanic | 2022 | 350 | 37391420 |
| Hispanic | 2023 | 365 | 38599156 |

**Supplemental Table 3f**: Population and Death Count by 10 year Age Groups from 1999-2023

| Ten-Year Age Groups | Year | Deaths | Population |
| --- | --- | --- | --- |
| 25-34 years | 1999 | 11 | 40178406 |
| 25-34 years | 2001 | 15 | 39471522 |
| 25-34 years | 2002 | 13 | 39349646 |
| 25-34 years | 2003 | 12 | 39243795 |
| 25-34 years | 2005 | 17 | 39258647 |
| 25-34 years | 2006 | 16 | 39395179 |
| 25-34 years | 2007 | 13 | 39713463 |
| 25-34 years | 2008 | 14 | 40207473 |
| 25-34 years | 2009 | 15 | 40723342 |
| 25-34 years | 2010 | 16 | 41063948 |
| 25-34 years | 2011 | 19 | 41790498 |
| 25-34 years | 2012 | 14 | 42309321 |
| 25-34 years | 2013 | 22 | 42844587 |
| 25-34 years | 2014 | 14 | 43516504 |
| 25-34 years | 2015 | 22 | 44137202 |
| 25-34 years | 2016 | 15 | 44677243 |
| 25-34 years | 2017 | 14 | 45342672 |
| 25-34 years | 2018 | 21 | 45697774 |
| 25-34 years | 2019 | 19 | 45940321 |
| 25-34 years | 2020 | 22 | 46069646 |
| 25-34 years | 2021 | 12 | 45495105 |
| 25-34 years | 2022 | 23 | 45501300 |
| 25-34 years | 2023 | 15 | 45542516 |
|  | | | |
| 35-44 years | 1999 | 45 | 45076677 |
| 35-44 years | 2000 | 53 | 45148527 |
| 35-44 years | 2001 | 50 | 45051752 |
| 35-44 years | 2002 | 51 | 44640649 |
| 35-44 years | 2003 | 46 | 44154206 |
| 35-44 years | 2004 | 45 | 43800275 |
| 35-44 years | 2005 | 39 | 43505538 |
| 35-44 years | 2006 | 47 | 43243801 |
| 35-44 years | 2007 | 32 | 42796230 |
| 35-44 years | 2008 | 55 | 42192486 |
| 35-44 years | 2009 | 60 | 41487811 |
| 35-44 years | 2010 | 57 | 41070606 |
| 35-44 years | 2011 | 34 | 40627954 |
| 35-44 years | 2012 | 35 | 40516420 |
| 35-44 years | 2013 | 32 | 40452690 |
| 35-44 years | 2014 | 49 | 40513133 |
| 35-44 years | 2015 | 45 | 40589783 |
| 35-44 years | 2016 | 54 | 40470156 |
| 35-44 years | 2017 | 53 | 40875370 |
| 35-44 years | 2018 | 46 | 41277888 |
| 35-44 years | 2019 | 55 | 41659144 |
| 35-44 years | 2020 | 47 | 42136192 |
| 35-44 years | 2021 | 39 | 43403854 |
| 35-44 years | 2022 | 54 | 43695365 |
| 35-44 years | 2023 | 64 | 44390693 |
|  | | | |
| 45-54 years | 1999 | 114 | 36577819 |
| 45-54 years | 2000 | 111 | 37677952 |
| 45-54 years | 2001 | 144 | 39386268 |
| 45-54 years | 2002 | 155 | 39992194 |
| 45-54 years | 2003 | 153 | 40819954 |
| 45-54 years | 2004 | 111 | 41629930 |
| 45-54 years | 2005 | 161 | 42495904 |
| 45-54 years | 2006 | 153 | 43286159 |
| 45-54 years | 2007 | 136 | 43939939 |
| 45-54 years | 2008 | 153 | 44460447 |
| 45-54 years | 2009 | 178 | 44867088 |
| 45-54 years | 2010 | 155 | 45006716 |
| 45-54 years | 2011 | 160 | 44718203 |
| 45-54 years | 2012 | 163 | 44268738 |
| 45-54 years | 2013 | 185 | 43767532 |
| 45-54 years | 2014 | 165 | 43458851 |
| 45-54 years | 2015 | 196 | 43188161 |
| 45-54 years | 2016 | 161 | 42786679 |
| 45-54 years | 2017 | 182 | 42374952 |
| 45-54 years | 2018 | 159 | 41631699 |
| 45-54 years | 2019 | 144 | 40874902 |
| 45-54 years | 2020 | 165 | 40366133 |
| 45-54 years | 2021 | 179 | 40688436 |
| 45-54 years | 2022 | 173 | 40431645 |
| 45-54 years | 2023 | 158 | 40493781 |
|  | | | |
| 55-64 years | 1999 | 255 | 23778026 |
| 55-64 years | 2000 | 246 | 24274684 |
| 55-64 years | 2001 | 257 | 25105295 |
| 55-64 years | 2002 | 253 | 26703332 |
| 55-64 years | 2003 | 283 | 28008945 |
| 55-64 years | 2004 | 287 | 29305304 |
| 55-64 years | 2005 | 293 | 30641497 |
| 55-64 years | 2006 | 303 | 31930113 |
| 55-64 years | 2007 | 343 | 33128434 |
| 55-64 years | 2008 | 311 | 34157063 |
| 55-64 years | 2009 | 335 | 35405600 |
| 55-64 years | 2010 | 354 | 36482729 |
| 55-64 years | 2011 | 417 | 38062140 |
| 55-64 years | 2012 | 363 | 38586202 |
| 55-64 years | 2013 | 378 | 39316431 |
| 55-64 years | 2014 | 414 | 40077581 |
| 55-64 years | 2015 | 405 | 40877819 |
| 55-64 years | 2016 | 444 | 41463144 |
| 55-64 years | 2017 | 424 | 41995658 |
| 55-64 years | 2018 | 433 | 42272636 |
| 55-64 years | 2019 | 420 | 42448537 |
| 55-64 years | 2020 | 460 | 42403677 |
| 55-64 years | 2021 | 468 | 42803064 |
| 55-64 years | 2022 | 453 | 42085437 |
| 55-64 years | 2023 | 421 | 41854411 |
|  | | | |
| 65-74 years | 1999 | 360 | 18418909 |
| 65-74 years | 2000 | 407 | 18390986 |
| 65-74 years | 2001 | 407 | 18384179 |
| 65-74 years | 2002 | 393 | 18388535 |
| 65-74 years | 2003 | 348 | 18500915 |
| 65-74 years | 2004 | 412 | 18667533 |
| 65-74 years | 2005 | 448 | 18881697 |
| 65-74 years | 2006 | 443 | 19203027 |
| 65-74 years | 2007 | 461 | 19698727 |
| 65-74 years | 2008 | 495 | 20505679 |
| 65-74 years | 2009 | 518 | 21233099 |
| 65-74 years | 2010 | 521 | 21713429 |
| 65-74 years | 2011 | 502 | 22481738 |
| 65-74 years | 2012 | 521 | 23985392 |
| 65-74 years | 2013 | 611 | 25216766 |
| 65-74 years | 2014 | 609 | 26398290 |
| 65-74 years | 2015 | 616 | 27550517 |
| 65-74 years | 2016 | 680 | 28630330 |
| 65-74 years | 2017 | 622 | 29683446 |
| 65-74 years | 2018 | 727 | 30492316 |
| 65-74 years | 2019 | 744 | 31483433 |
| 65-74 years | 2020 | 781 | 32549398 |
| 65-74 years | 2021 | 857 | 33666122 |
| 65-74 years | 2022 | 829 | 33788439 |
| 65-74 years | 2023 | 890 | 34685284 |
|  | | | |
| 75-84 years | 1999 | 463 | 12224914 |
| 75-84 years | 2000 | 493 | 12361180 |
| 75-84 years | 2001 | 516 | 12593618 |
| 75-84 years | 2002 | 550 | 12764864 |
| 75-84 years | 2003 | 513 | 12896438 |
| 75-84 years | 2004 | 540 | 12989903 |
| 75-84 years | 2005 | 551 | 13074802 |
| 75-84 years | 2006 | 585 | 13095151 |
| 75-84 years | 2007 | 620 | 13087439 |
| 75-84 years | 2008 | 630 | 13076102 |
| 75-84 years | 2009 | 595 | 13022775 |
| 75-84 years | 2010 | 615 | 13061122 |
| 75-84 years | 2011 | 611 | 13175230 |
| 75-84 years | 2012 | 601 | 13272634 |
| 75-84 years | 2013 | 644 | 13446519 |
| 75-84 years | 2014 | 607 | 13682690 |
| 75-84 years | 2015 | 681 | 13923174 |
| 75-84 years | 2016 | 742 | 14233534 |
| 75-84 years | 2017 | 706 | 14706551 |
| 75-84 years | 2018 | 730 | 15394374 |
| 75-84 years | 2019 | 819 | 15969872 |
| 75-84 years | 2020 | 797 | 16451547 |
| 75-84 years | 2021 | 823 | 16206075 |
| 75-84 years | 2022 | 953 | 17520545 |
| 75-84 years | 2023 | 955 | 18368097 |
|  | | | |
| 85+ years | 1999 | 270 | 4154018 |
| 85+ years | 2000 | 294 | 4239587 |
| 85+ years | 2001 | 262 | 4312494 |
| 85+ years | 2002 | 265 | 4368808 |
| 85+ years | 2003 | 268 | 4466176 |
| 85+ years | 2004 | 336 | 4545883 |
| 85+ years | 2005 | 326 | 4693299 |
| 85+ years | 2006 | 349 | 4865929 |
| 85+ years | 2007 | 357 | 5039545 |
| 85+ years | 2008 | 387 | 5195840 |
| 85+ years | 2009 | 394 | 5367301 |
| 85+ years | 2010 | 426 | 5493433 |
| 85+ years | 2011 | 458 | 5737173 |
| 85+ years | 2012 | 433 | 5887330 |
| 85+ years | 2013 | 477 | 6040789 |
| 85+ years | 2014 | 482 | 6162231 |
| 85+ years | 2015 | 483 | 6287161 |
| 85+ years | 2016 | 515 | 6380331 |
| 85+ years | 2017 | 492 | 6468682 |
| 85+ years | 2018 | 564 | 6544503 |
| 85+ years | 2019 | 556 | 6604958 |
| 85+ years | 2020 | 598 | 6658420 |
| 85+ years | 2021 | 650 | 5975756 |
| 85+ years | 2022 | 648 | 6485868 |
| 85+ years | 2023 | 627 | 6194980 |

**Supplemental Table 3g**: Population and Death Count by Region from 1999-2023

| Census Region | Year | Deaths | Population |
| --- | --- | --- | --- |
| Northeast | 1999 | 341 | 35633134 |
| Northeast | 2000 | 379 | 35788687 |
| Northeast | 2001 | 350 | 36006250 |
| Northeast | 2002 | 339 | 36185082 |
| Northeast | 2003 | 341 | 36346948 |
| Northeast | 2004 | 364 | 36462699 |
| Northeast | 2005 | 361 | 36559788 |
| Northeast | 2006 | 381 | 36682176 |
| Northeast | 2007 | 404 | 36846338 |
| Northeast | 2008 | 395 | 37084149 |
| Northeast | 2009 | 425 | 37339597 |
| Northeast | 2010 | 451 | 37543347 |
| Northeast | 2011 | 429 | 37864117 |
| Northeast | 2012 | 397 | 38158527 |
| Northeast | 2013 | 467 | 38437194 |
| Northeast | 2014 | 423 | 38710627 |
| Northeast | 2015 | 448 | 38965872 |
| Northeast | 2016 | 450 | 39040202 |
| Northeast | 2017 | 476 | 39417175 |
| Northeast | 2018 | 484 | 39321978 |
| Northeast | 2019 | 511 | 39381333 |
| Northeast | 2020 | 515 | 39418559 |
| Northeast | 2021 | 453 | 40339548 |
| Northeast | 2022 | 540 | 40289184 |
| Northeast | 2023 | 517 | 40402747 |
|  | | | |
| Midwest | 1999 | 358 | 41293967 |
| Midwest | 2000 | 412 | 41504992 |
| Midwest | 2001 | 377 | 41762990 |
| Midwest | 2002 | 410 | 41970731 |
| Midwest | 2003 | 400 | 42200881 |
| Midwest | 2004 | 414 | 42455980 |
| Midwest | 2005 | 421 | 42748579 |
| Midwest | 2006 | 458 | 43084311 |
| Midwest | 2007 | 447 | 43424366 |
| Midwest | 2008 | 452 | 43718509 |
| Midwest | 2009 | 472 | 44010460 |
| Midwest | 2010 | 474 | 44248465 |
| Midwest | 2011 | 514 | 44584105 |
| Midwest | 2012 | 482 | 44817227 |
| Midwest | 2013 | 519 | 45090597 |
| Midwest | 2014 | 512 | 45360409 |
| Midwest | 2015 | 531 | 45628315 |
| Midwest | 2016 | 577 | 45802491 |
| Midwest | 2017 | 517 | 46143783 |
| Midwest | 2018 | 571 | 46405110 |
| Midwest | 2019 | 631 | 46589364 |
| Midwest | 2020 | 620 | 46721679 |
| Midwest | 2021 | 665 | 47032092 |
| Midwest | 2022 | 701 | 47087294 |
| Midwest | 2023 | 637 | 47312396 |
|  | | | |
| South | 1999 | 483 | 64108630 |
| South | 2000 | 493 | 64843390 |
| South | 2001 | 584 | 65874012 |
| South | 2002 | 548 | 66758178 |
| South | 2003 | 518 | 67639133 |
| South | 2004 | 571 | 68718205 |
| South | 2005 | 626 | 69951038 |
| South | 2006 | 605 | 71198336 |
| South | 2007 | 608 | 72387467 |
| South | 2008 | 695 | 73529724 |
| South | 2009 | 684 | 74596130 |
| South | 2010 | 735 | 75419767 |
| South | 2011 | 729 | 76650163 |
| South | 2012 | 714 | 77681893 |
| South | 2013 | 793 | 78693557 |
| South | 2014 | 801 | 79945474 |
| South | 2015 | 840 | 81260812 |
| South | 2016 | 904 | 82405493 |
| South | 2017 | 869 | 83659214 |
| South | 2018 | 913 | 84717024 |
| South | 2019 | 931 | 85606773 |
| South | 2020 | 993 | 86611804 |
| South | 2021 | 1115 | 86926028 |
| South | 2022 | 1071 | 87978320 |
| South | 2023 | 1195 | 89219544 |
|  | | | |
| West | 1999 | 336 | 39373038 |
| West | 2000 | 328 | 39847571 |
| West | 2001 | 340 | 40661876 |
| West | 2002 | 383 | 41294037 |
| West | 2003 | 364 | 41903467 |
| West | 2004 | 391 | 42568500 |
| West | 2005 | 427 | 43291979 |
| West | 2006 | 452 | 44054536 |
| West | 2007 | 503 | 44745606 |
| West | 2008 | 503 | 45462708 |
| West | 2009 | 514 | 46160829 |
| West | 2010 | 484 | 46680404 |
| West | 2011 | 529 | 47494551 |
| West | 2012 | 537 | 48168390 |
| West | 2013 | 570 | 48863966 |
| West | 2014 | 604 | 49792770 |
| West | 2015 | 629 | 50698818 |
| West | 2016 | 680 | 51393231 |
| West | 2017 | 631 | 52227159 |
| West | 2018 | 712 | 52867078 |
| West | 2019 | 684 | 53403697 |
| West | 2020 | 742 | 53882971 |
| West | 2021 | 795 | 53940744 |
| West | 2022 | 821 | 54153801 |
| West | 2023 | 781 | 54595075 |
